# Longitudinal joint trajectories of the dietary inflammatory index and physical activity and risk of incident cardiometabolic multimorbidity among women: evidence from the China Health and Nutrition Survey

**DOI:** 10.3389/fpubh.2026.1916853

**Published:** 2026-10-20

**Authors:** Shaoxing Li, Xiangjie Liu, Kai Gong, Yu Tian, Hua Yu

**Affiliations:** 1 Department of Obstetrics and Gynecology, The First Affiliated Hospital of Chengdu Medical College, Chengdu, Sichuan, China; 2 Department of Orthopedics, The First Affiliated Hospital of Chengdu Medical College, Chengdu, Sichuan, China; 3 Department of Dermatology and Venereology, The First Affiliated Hospital of Chengdu Medical College, Chengdu, Sichuan, China

**Keywords:** adult women, cardiometabolic multimorbidity, dietary inflammatory index, joint trajectory, longitudinal study, physical activity

## Abstract

**Objective:**

To examine the association between long-term joint trajectories of the dietary inflammatory index (DII) and physical activity (PA) and the risk of incident cardiometabolic multimorbidity (CMM) among Chinese women.

**Methods:**

Data were drawn from the China Health and Nutrition Survey 2004–2015. A total of 3,970 adult women without CMM at baseline were included. DII and PA were repeatedly assessed from 2004 to 2011, and joint trajectories were identified via group-based trajectory modeling. CMM was defined as the first co-occurrence of ≥2 of hypertension, diabetes, stroke, and heart disease. Cox models evaluated associations between trajectory and incident CMM, with subgroup and sensitivity analyses performed.

**Results:**

During follow-up, 400 participants (10.1%) developed incident CMM. Three joint trajectories were identified: lower DII + moderately active (*n* = 559, 14.1%), moderate DII + highly active (*n* = 541, 13.6%), and higher DII + inactive (*n* = 2,870, 72.3%). Compared with the lower DII + moderately active group, higher DII + inactive group showed significantly higher CMM risk (HR = 1.62, 95% CI: 1.14–2.31; *p* = 0.007), whereas the moderate DII + highly active group did not. No significant effect modification was observed across subgroups. The association persisted in sensitivity analyses.

**Conclusion:**

A sustained pattern of higher dietary inflammation with lower physical activity was associated with elevated incident CMM risk in Chinese women, providing longitudinal evidence for earlier risk identification based on behavioral exposures.

## Graphical abstract

**Figure:**
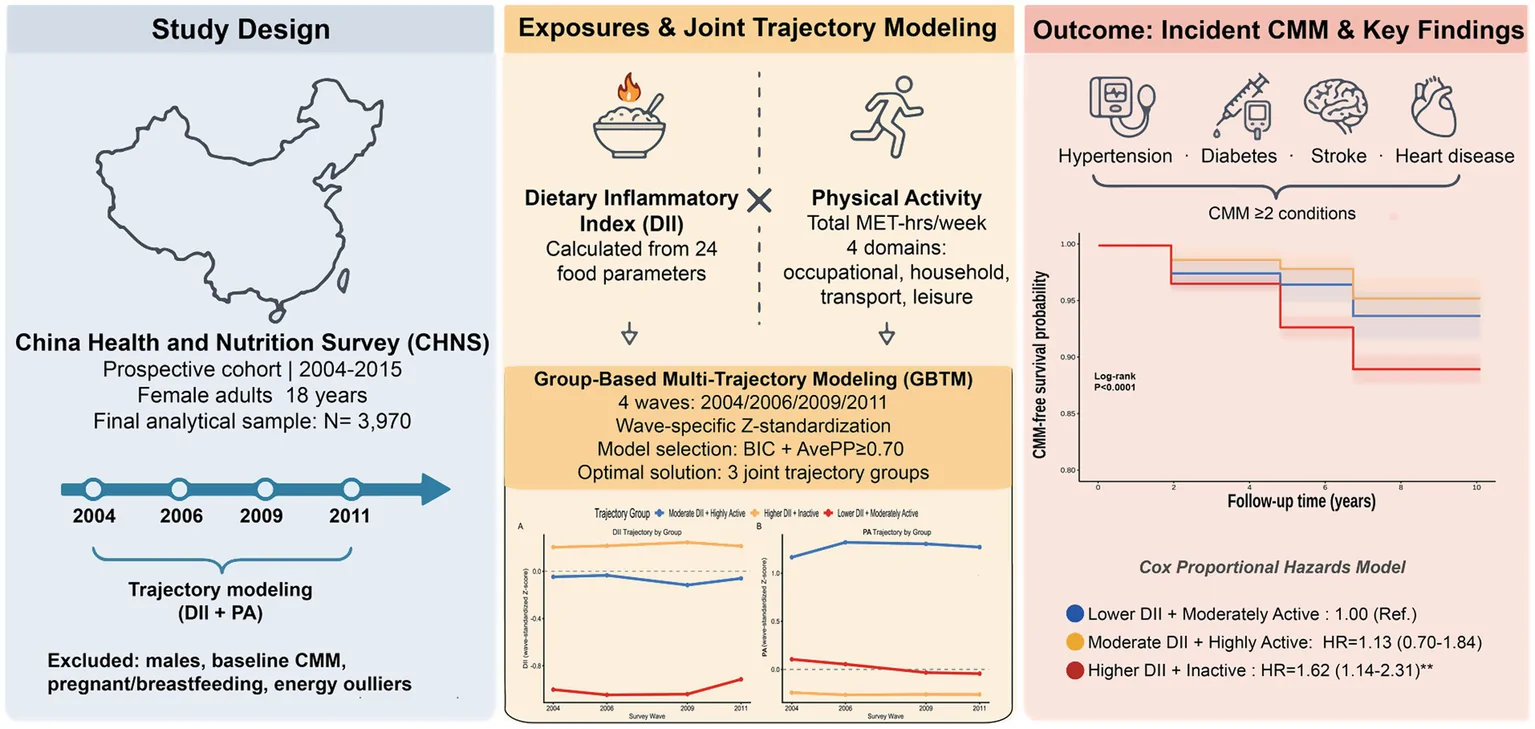

## Introduction

Cardiometabolic multimorbidity (CMM), defined as the coexistence of at least two of diabetes, hypertension, heart disease, and stroke, reflects the cumulative burden of overlapping risk factors and etiologic pathways (1). Among middle-aged and older Chinese women, CMM represents a substantial health burden and is closely associated with adverse health outcomes (2). Women experience unique physiological transitions, including pregnancy and menopause, which interact with social and behavioral factors to shape distinctive trajectories of risk accumulation (3). However, current chronic disease prevention strategies remain largely disease-specific and are poorly suited to address the complex needs of individuals with multimorbidity, including polypharmacy, fragmented care, and amplified functional impairment; female-specific prevention strategies are also limited (4, 5). Once CMM develops, clinical priorities often shift from primary prevention to multimorbidity management and complication control, substantially narrowing the window for early intervention (6). Compared with single cardiometabolic diseases, CMM is associated with greater clinical complexity, higher functional burden, increased healthcare utilization, and elevated mortality risk (7, 8). Against the background of a growing chronic disease burden, identifying long-term risk factors and exposure patterns before the onset of CMM is essential for early prevention.

Lifestyle intervention is a low-cost and accessible strategy across the continuum of CMM prevention and management, and represents an important entry point for primary prevention and early risk identification (6). Diet and physical activity (PA) are two core behavioral dimensions that reflect energy intake and expenditure, respectively. The dietary inflammatory index (DII), a comprehensive measure of the inflammatory potential of the overall diet, may promote the clustering of obesity, insulin resistance, dyslipidemia, and hypertension through chronic low-grade inflammation, thereby contributing to the progression toward CMM (9, 10). PA, which captures habitual activity level and energy expenditure, also modulates these metabolic disturbances and is closely linked to the dynamic progression of CMM (11). These associations may be particularly relevant during female-specific physiological transitions. For example, elevated estrogen levels during pregnancy are accompanied by changes in central adiposity, glucose metabolism, and lipid profiles, which may influence cardiometabolic risk over the subsequent decade (12). Regular exercise, by contrast, has been shown to improve cardiometabolic health among postmenopausal women (13). Therefore, characterizing long-term exposure patterns of DII and PA in adult women may provide early behavioral warning signals for early CMM prevention in women.

Previous studies, mostly based on single measurements in general populations, have linked DII or PA to cardiovascular disease, metabolic syndrome, and CMM-related outcomes (11, 14–16). However, limited attention has been paid to the long-term accumulation of risk and its progression to multimorbidity among Chinese women, particularly in the context of population aging, nutrition transition, and urban–rural disparities. One study using data from 1993 to 2004 examined the long-term joint patterns of diet quality and physical activity in relation to outcomes such as diabetes (17), suggesting that combined lifestyle patterns may contribute to the development of cumulative outcomes such as CMM. Nevertheless, longitudinal evidence on the joint trajectories of DII and PA and their association with incident CMM among Chinese adult women remains scarce. The China Health and Nutrition Survey (CHNS) has systematically collected long-term data on diet, physical activity, and health outcomes, providing a robust basis for characterizing longitudinal exposure patterns (16). Using CHNS data from 2004 to 2015, this study focused on adult women and applied a multivariable group-based trajectory model to four repeated measurements of DII and PA. We further evaluated the association between distinct joint trajectory groups and the risk of incident CMM, aiming to provide longitudinal population-based evidence for early risk identification before the onset of CMM among Chinese women.

## Materials and methods

### Data source

Data were obtained from the CHNS, an open, prospective longitudinal cohort study jointly conducted by the Carolina Population Center at the University of North Carolina at Chapel Hill and the National Institute for Nutrition and Health of the Chinese Center for Disease Control and Prevention (16). Initiated in 1989, the CHNS used a multistage, random cluster sampling design to recruit participants from provinces and cities across different geographic regions and socioeconomic strata in China. The survey systematically collects information on sociodemographic characteristics, dietary intake, physical activity, lifestyle factors, and health outcomes.

This study used data from five survey waves conducted in 2004, 2006, 2009, 2011, and 2015. The year 2004 was defined as the study baseline and the start of follow-up, while 2006 was the first post-baseline wave at which incident CMM could be ascertained. Repeated measurements of the DII and PA from 2004 to 2011 were used to construct long-term joint trajectories, and CMM status was followed through 2015. The exposure assessment began in 2004 because, from 2004 onward, nutrient calculations in the CHNS were consistently based on the 2002 Chinese Food Composition Table, thereby improving the comparability of DII estimation across waves. The CHNS protocol was approved by the relevant institutional review boards, and the present study used publicly available de-identified CHNS data and required no additional ethical approval or informed consent.

### Study population

This study was restricted to adult female participants in the 2004 CHNS survey. Participants were included if they met the following criteria: (1) age ≥18 years at the 2004 baseline; (2) complete DII and PA data in at least two survey waves between 2004 and 2011; and (3) at least one post-baseline assessment of CMM status. Participants were excluded if they (1) were pregnant or lactating at baseline; (2) had prevalent CMM at baseline; or (3) had implausible total energy intake (<600 or >4,000 kcal/day). The participant selection process is shown in Figure 1.

**Figure 1:**
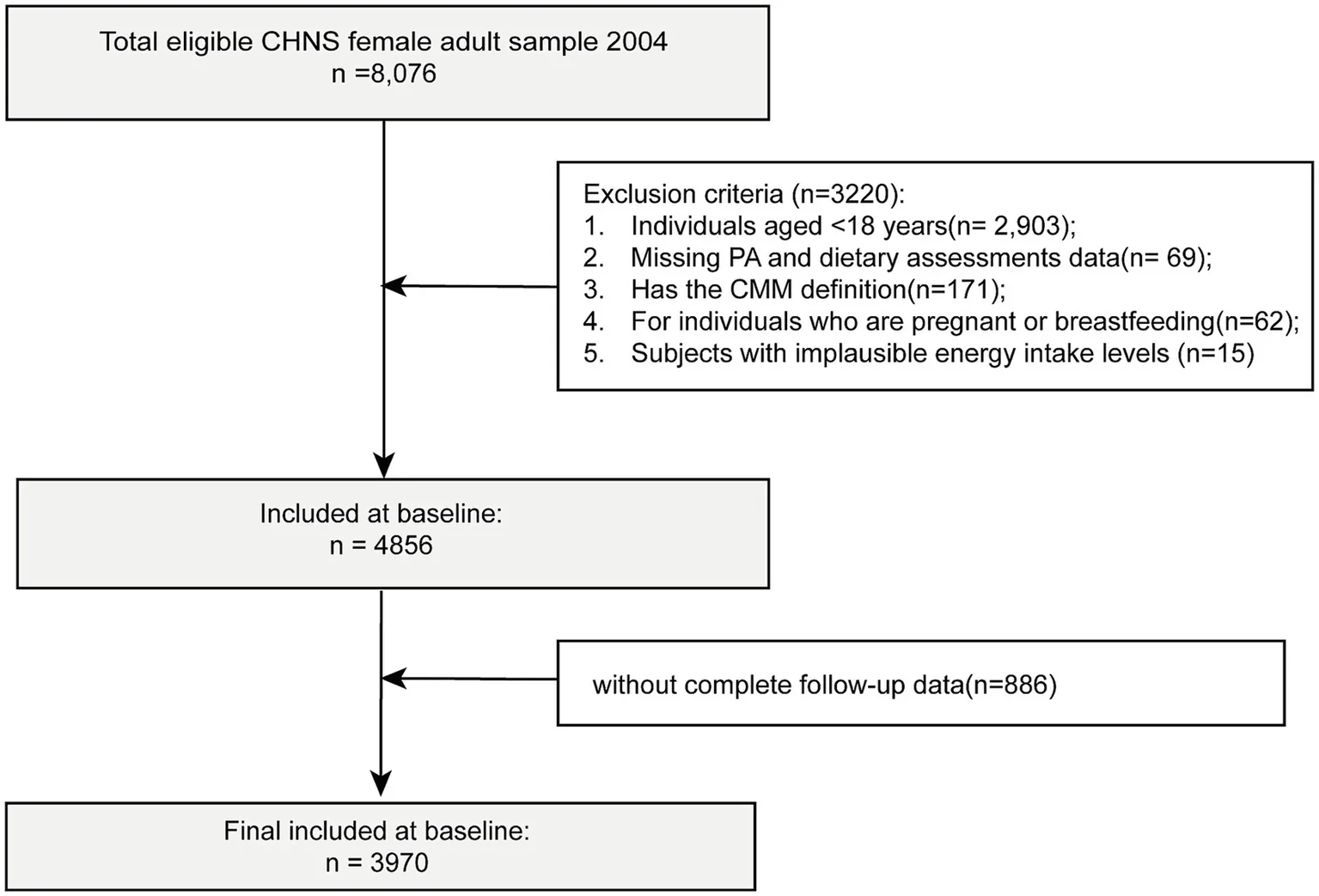
Flowchart of participant selection. CHNS, China Health and Nutrition Survey; PA, physical activity; CMM, cardiometabolic multimorbidity.

### Dietary assessment

Dietary intake was assessed in each CHNS wave using a combination of three consecutive 24-h dietary recalls and household food-weighing records. The three recall days generally included two weekdays and one weekend day. Trained interviewers conducted face-to-face interviews to record all foods and beverages consumed during the preceding 24 h, including food type, amount, and cooking method. Food models, photographs, and other visual aids were used to improve the accuracy of portion-size estimation. Household food-weighing records were used to supplement information on household-level consumption of edible oils, condiments, and shared foods. Individual average daily nutrient intake was calculated by matching food consumption data with the 2002 Chinese Food Composition Table.

### Calculation of the dietary inflammatory index

The DII was calculated according to the methodological framework developed by Shivappa et al. (18). Based on the availability of dietary parameters in the CHNS and the Chinese Food Composition Table, 24 dietary parameters were included in the present study. These comprised 18 nutrient-level parameters: energy (kcal), protein (g), total fat (g), carbohydrate (g), dietary fiber (g), cholesterol (mg), vitamin A (μg retinol equivalents), β-carotene (μg), vitamin C (mg), thiamine (mg), riboflavin (mg), niacin (mg), vitamin E (mg), iron (mg), magnesium (mg), zinc (mg), selenium (μg), and alcohol (g); and six food-level parameters: garlic (g), ginger (g), onion (g), green/black tea (g), chili pepper (g), and soy isoflavones (mg). Food-level parameters were derived from household food-weighing records in the nutr1 file by identifying relevant food items and aggregating daily consumption. Soy isoflavone intake was estimated from soybean product intake using an average isoflavone content of 0.5 mg/g. The global reference means, standard deviations, inflammatory effect scores, and available survey waves for each parameter are shown in Supplementary Table S1.

The DII was calculated in four steps. First, the average daily intake of each parameter was standardized to a Z-score using the corresponding global reference mean and standard deviation: Z = (individual intake − global mean)/global standard deviation. Second, each Z score was converted to a centered percentile using the standard normal cumulative distribution function: centered percentile = Φ(Z) × 2–1, yielding values from −1 to +1. Third, the centered percentile for each parameter was multiplied by its literature-derived inflammatory effect score. Finally, the 24 parameter-specific DII scores were summed to obtain the overall DII score for each participant. Energy was included as one of the 24 DII parameters and was processed using the same global standardization and weighting procedure as the other parameters. A higher DII score indicated a more pro-inflammatory diet, whereas a lower score indicated a more anti-inflammatory diet.

### Assessment of physical activity

PA levels were calculated based on previously published methods (19). PA was assessed in each survey wave using interviewer-administered questionnaires covering four domains: occupational activity, household activity, transportation activity, and leisure-time activity. Participants reported the average frequency and duration of each type of activity during the preceding year. Metabolic equivalent (MET) values, defined as the ratio of activity-related metabolic rate to resting metabolic rate, were assigned according to the Compendium of Physical Activities. For each activity, weekly duration was multiplied by the corresponding MET value to calculate MET-hours per week (MET-h/week).

Occupational activity was classified according to self-reported job content as light, moderate, or heavy activity, corresponding to MET values of 2.0, 4.0, and 6.0, respectively. Light activity referred to sedentary work or work involving occasional standing; moderate activity included occupations such as drivers and electricians; and heavy activity included farmers, athletes, dancers, steel workers, loggers, and construction workers. Household activity was assessed based on five activities: food purchasing, food preparation or cooking, laundry, house cleaning, and childcare. Transportation activity was assessed according to four commuting modes: walking, cycling, taking a bus, and driving. Leisure-time activity included martial arts, gymnastics or dancing, jogging or swimming, ball games such as football, basketball, tennis, badminton, volleyball, and table tennis, and other activities. Total PA was defined as the sum of MET-h/week across occupational, household, transportation, and leisure-time activities.

To minimize the influence of outliers, all time-related variables were cleaned before calculation. Values coded as −99, indicating unknown or refusal to answer, were set as missing, and physiologically implausible extreme duration values were truncated.

### Outcome definition

The primary outcome was incident CMM. CMM was defined as the coexistence of at least two of four cardiometabolic diseases: hypertension, diabetes, stroke, and heart disease. Hypertension, diabetes, and stroke were mainly ascertained by self-reported physician diagnoses, whereas heart disease was identified using the available CHNS heart disease and myocardial infarction items across survey waves. Participants who did not meet the definition of CMM at baseline, that is, those with none or only one of the above diseases, but subsequently developed at least two coexisting diseases during follow-up were classified as having incident CMM. Chronic disease status was treated cumulatively; once a component disease was identified in any survey wave, it was considered to persist in subsequent waves. Because exact onset dates between survey waves were unavailable, event time was assigned to the time point of the first survey wave at which CMM was identified. Follow-up time was calculated from the 2004 baseline to the first occurrence of CMM, death, loss to follow-up, or the last survey in 2015, whichever occurred first. Detailed definitions and source variables are provided in Supplementary Table S2.

### Covariates

The selection of covariates was based on existing literature evidence in the CMM field and the availability of CHNS data. The following baseline covariates were ultimately included: age (continuous variable), place of residence (urban/rural), education level (elementary school or below/middle school/high school or above), marital status (married/cohabiting/others), smoking status (never/drew ever/currently), alcohol consumption status (never/drew ever/currently), body mass index (BMI), systolic blood pressure (SBP) and diastolic blood pressure (DBP) (continuous, mmHg; calculated as the mean of at least two valid measurements at baseline), and baseline CMM component disease status (0 or 1 component condition).

### Handling of missing data

Trajectory analysis included participants with DII and PA data available in at least two survey waves. Under the missing-at-random assumption, intermittently missing pre-event DII and PA measurements were handled using the maximum likelihood method of a latent class mixture model. Outcome data and covariate data were analyzed using complete data. Data availability was summarized by survey wave, and baseline characteristics were compared between participants included in the final analysis and those excluded because of incomplete follow-up or insufficient exposure data.

### Statistical analysis

#### Descriptive analysis

Continuous variables were summarized as means with standard deviations or medians with interquartile ranges, according to their distribution. Categorical variables were presented as counts and percentages. Baseline characteristics were compared according to incident CMM status. Student’s t test or the Wilcoxon rank-sum test was used for continuous variables, and the χ^2^ test was used for categorical variables. Ridge plots were used to display the kernel density distributions of DII and PA in 2004, 2006, 2009, and 2011, describing the distributional features of repeated exposures across waves.

#### Baseline exposure analysis

Baseline DII and PA levels in 2004 were each categorized into tertiles, and Cox proportional hazards models were used to examine the associations of individual exposure levels with incident CMM. In addition, baseline DII and PA were dichotomized at their respective medians and cross-classified into four joint exposure groups: lower DII + active, lower DII + inactive, higher DII + active, and higher DII + inactive. Using the lower DII + active group as the reference, Cox proportional hazards models were used to estimate hazard ratios (HRs) and 95% confidence intervals (CIs) for incident CMM. Models were adjusted for age, residence area, educational level, marital status, BMI, SBP, DBP, baseline CMM component disease status, smoking status, and drinking status.

Within the baseline joint exposure framework, additive interaction between DII and PA was assessed using relative excess risk due to interaction (RERI), attributable proportion due to interaction (AP), and the synergy index (SI). Confidence intervals were estimated using bootstrap resampling.

Restricted cubic spline (RCS) analyses were performed to examine potential nonlinear associations of DII and PA with incident CMM. Knots were placed at the 5th, 35th, 65th, and 95th percentiles.

#### Joint trajectory modeling of DII and physical activity

Multivariable group-based trajectory modeling (GBTM) (20) was used to identify long-term joint patterns of DII and PA across 2004, 2006, 2009, and 2011. For participants who developed CMM during this period, only DII and PA measurements obtained before the survey wave in which CMM was first identified were included in trajectory estimation; measurements from the event wave and subsequent waves were excluded. GBTM assumes that the study population comprises several latent subgroups with distinct longitudinal exposure patterns and estimates both group-specific trajectory shapes and individual posterior probabilities of group membership using a finite mixture model. In the present study, DII and PA were modeled simultaneously as two parallel longitudinal processes sharing the same latent class structure, thereby identifying trajectory groups that represented joint longitudinal patterns of relative DII and PA levels.

Because DII and PA were measured on different scales, both variables were standardized to Z scores within each survey wave before trajectory modeling, with a mean of 0 and a standard deviation of 1 (21). This approach allowed the trajectory model to reflect each participant’s relative position within the sample at each wave and reduced the influence of differences in numerical scale on model fitting. For DII, a higher Z score indicated a relatively more pro-inflammatory dietary pattern, whereas for PA, a higher Z score indicated a relatively higher activity level. Trajectory groups were named according to the relative DII and PA levels represented by their fitted trajectories across waves. After trajectory classification, the original, unstandardized DII and PA values were used to describe the exposure profiles of each trajectory group, while trajectory group membership was used as the exposure in subsequent Cox regression analyses.

The number of trajectory groups was iteratively tested from two to six, and polynomial functions from first-order linear to third-order cubic forms were compared. The final model was selected by (1) jointly considering the Akaike information criterion (AIC) and the Bayesian information criterion (BIC), (2) the average posterior probability (AvePP) ≥ 0.70 for each group; (3) a minimum group proportion of 5%; and (4) the interpretability of the resulting trajectory patterns. Each participant was assigned to the trajectory group with the highest posterior probability. A random seed of 42 was used to ensure reproducibility. Principal component analysis (PCA) biplots were used to reduce the dimensionality of multi-wave DII and PA data and visualize the distribution and separation of trajectory groups in the multidimensional exposure space.

#### Joint trajectory groups and risk of incident CMM

Cox proportional hazards models were used to evaluate the association between DII–PA joint trajectory groups and incident CMM. The lower DII + moderately active group was used as the reference, and HRs with 95% CIs were calculated for each trajectory group. A sequential adjustment strategy was applied. Model 1 included only trajectory group. Model 2 was further adjusted for age, residence area, educational level, and marital status. Model 3 was additionally adjusted for BMI, SBP, DBP, baseline CMM component disease status, smoking status and drinking status. Kaplan–Meier curves were used to display CMM-free survival probabilities across joint trajectory groups, and between-group differences were assessed using the log-rank test.

The proportional hazards assumption for the fully adjusted Cox model was assessed using Schoenfeld residuals. Both global and variable-specific *p* values were reported. If the residual tests were not statistically significant, no clear evidence of violation of the proportional hazards assumption was considered to be present.

#### Subgroup analysis

Subgroup analyses were conducted according to age (<50 or ≥50 years), educational level (primary school or below, middle school, or high school or above), marital status (married/cohabiting or other), and baseline CMM component disease status (0 or 1 component condition). Fully adjusted Cox models, corresponding to Model 3, were repeated within each subgroup. Effect modification was tested by adding multiplicative interaction terms between trajectory group and the corresponding stratification variable to the model.

#### Sensitivity analysis

Three sensitivity analyses were conducted. First, Cox regression analyses were repeated after excluding participants who developed CMM within the first 2 years after the start of follow-up to reduce the potential influence of reverse causation. Second, Fine–Gray competing risk models were fitted with death as a competing event, and subdistribution hazard ratios (SHRs) were reported to assess the influence of competing mortality risk on the main findings. Third, the primary Cox analysis was repeated after excluding participants with any CMM component disease at baseline, restricting the analysis to participants free of all four component diseases at study entry. Sensitivity models used the Model 3 covariate set; baseline CMM component disease status was omitted from the disease-free analysis because it was invariant in this restricted sample.

#### Statistical software

All statistical analyses were performed using R software. Ridge plots were generated using the ggridges package. Cox regression models were fitted using the survival package, and Schoenfeld residual tests were performed using the cox.zph() function. RERI and related additive interaction indices were calculated using the interactionR package or by manual calculation. Joint trajectory modeling was performed using the lcmm package. PCA analyses were conducted using the FactoMineR and factoextra packages. Fine–Gray competing risk models were fitted using the cmprsk or tidycmprsk package. Kaplan–Meier curves were generated using the survminer package. All statistical tests were two-sided, and *p* < 0.05 was considered statistically significant.

## Results

### Study population and baseline characteristics

After screening, 3,970 participants were included in the final analysis (Figure 1). Most were middle-aged, married or cohabiting women, and 89.8% had no CMM component disease at baseline (Table 1). During a mean follow-up of 8.2 ± 3.4 years, 400 incident CMM cases occurred, corresponding to a cumulative incidence of 10.1%.

**Table 1:** Baseline characteristics of study participants according to incident CMM status.

Variable	Total (*N* = 3,970)	No CMM (*n* = 3,570)	CMM (*n* = 400)	*p* value
Age, years	48.5 ± 14.2	47.8 ± 14.0	54.7 ± 14.4	<0.001
BMI, kg/m^2^	23.2 ± 3.4	23.1 ± 3.3	24.7 ± 4.0	<0.001
WHtR	0.5 ± 0.1	0.5 ± 0.1	0.5 ± 0.1	<0.001
Systolic BP, mmHg	120.6 ± 19.4	119.9 ± 18.9	127.5 ± 22.0	<0.001
Diastolic BP, mmHg	77.4 ± 11.5	77.1 ± 11.4	80.1 ± 11.7	<0.001
total_MET, MET-h/wk	136.2 ± 138.3	140.5 ± 139.5	97.9 ± 121.2	<0.001
DII score	3.0 ± 1.4	3.0 ± 1.5	3.4 ± 1.3	<0.001
Energy, kcal/d	2030.7 ± 583.6	2042.9 ± 581.7	1921.2 ± 589.7	<0.001
Protein, g/d	61.7 ± 21.1	62.1 ± 21.2	57.9 ± 20.3	<0.001
Fat, g/d	64.9 ± 34.7	65.1 ± 34.6	63.3 ± 35.7	0.179
Fiber, g/d	11.3 ± 6.6	11.4 ± 6.7	10.3 ± 5.4	0.007
Vitamin C, mg/d	94.9 ± 58.7	95.9 ± 59.1	85.6 ± 54.4	<0.001
Vitamin E, mg/d	11.4 ± 7.3	11.5 ± 7.4	10.6 ± 6.6	0.039
Selenium, μg/d	41.4 ± 22.3	41.6 ± 22.4	39.9 ± 22.2	0.060
Zinc, mg/d	10.7 ± 4.1	10.8 ± 4.1	9.7 ± 3.6	<0.001
Follow-up, years	8.2 ± 3.4	8.5 ± 3.3	5.7 ± 3.1	<0.001
Education				
High school or above	726 (18.3%)	671 (18.8%)	55 (13.8%)	<0.001
Middle school	1,038 (26.1%)	966 (27.1%)	72 (18.0%)	
Primary or below	2,206 (55.6%)	1933 (54.1%)	273 (68.2%)	
Region				
Urban	1,351 (34.0%)	1,210 (33.9%)	141 (35.2%)	0.626
Rural	2,619 (66.0%)	2,360 (66.1%)	259 (64.8%)	
Marital status				
Married/cohabiting	3,382 (85.2%)	3,059 (85.7%)	323 (80.8%)	0.010
Other	588 (14.8%)	511 (14.3%)	77 (19.2%)	
Smoking status				
Never	3,790 (95.5%)	3,414 (95.6%)	376 (94.0%)	0.174
Former/current	180 (4.5%)	156 (4.4%)	24 (6.0%)	
Drinking status				
Never/former	3,636 (91.6%)	3,261 (91.3%)	375 (93.8%)	0.121
Current	334 (8.4%)	309 (8.7%)	25 (6.2%)	
Baseline CMM component disease				
0	3,567 (89.8%)	3,228 (90.4%)	339 (84.8%)	<0.001
1	403 (10.2%)	342 (9.6%)	61 (15.2%)	

Continuous variables are presented as means ± standard deviations, and categorical variables are presented as numbers with percentages. CMM, cardiometabolic multimorbidity; DII, dietary inflammatory index; BMI, body mass index; WHtR, waist-to-height ratio; MET, metabolic equivalent.

Compared with participants who did not develop CMM, those with incident CMM were older (54.7 ± 14.4 years vs. 47.8 ± 14.0 years, *p* < 0.001), were more likely to have primary school education or below (68.2% vs. 54.1%, *p* < 0.001), had higher BMI, SBP, DBP, and were more likely to have one CMM component disease at baseline (all *p* < 0.001). In addition, they had lower PA levels (97.9 ± 121.2 vs. 140.5 ± 139.5 MET-h/week, *p* < 0.001), higher DII scores (3.4 ± 1.3 vs. 3.0 ± 1.5, *p* < 0.001), and lower intakes of total energy, protein, dietary fiber, vitamin C, vitamin E, and zinc (all *p* < 0.05).

Regarding data completeness, data availability decreased across successive follow-up waves (Supplementary Table S3). Included and excluded participants were comparable in smoking, drinking, and baseline CMM component disease status, whereas differences were observed in age, BMI, blood pressure, sociodemographic characteristics, DII, and PA (Supplementary Table S4).

### Associations of baseline DII and PA exposures with incident CMM

Baseline DII and PA were associated with the risk of incident CMM. Tertile-based analyses showed that higher DII was associated with increased CMM risk, whereas higher PA was associated with lower risk (Supplementary Table S5). In the median-based joint exposure analysis, participants in the higher DII + inactive group had the highest risk of incident CMM (HR = 1.81, 95% CI: 1.32 to 2.46). However, no significant additive interaction was observed between DII and PA (RERI = −0.164, 95% CI: −0.886 to 0.376; AP = −0.091, 95% CI: −0.478 to 0.212; SI = 0.831, 95% CI: 0.439 to 1.915) (Supplementary Table S6). RCS analyses further showed a positive linear association between DII and incident CMM risk (*p* for overall < 0.001; *p* for nonlinearity = 0.204), whereas total PA showed a negative linear association with CMM risk (*p* for overall = 0.006; *p* for nonlinearity = 0.356) (Supplementary Figure S1). These findings supported the subsequent evaluation of long-term joint DII–PA patterns.

### Multi-wave distributions of DII and PA

Across the four survey waves in 2004, 2006, 2009, and 2011, DII scores were mainly concentrated in the positive range and showed an increasing trend over time (Figure 2A), suggesting a generally pro-inflammatory dietary pattern. The distribution of PA was markedly right-skewed, with most participants clustered at low-to-moderate PA levels and a small proportion showing high activity levels (Figure 2B). Both indicators showed substantial inter-individual heterogeneity and temporal variation during follow-up.

**Figure 2:**
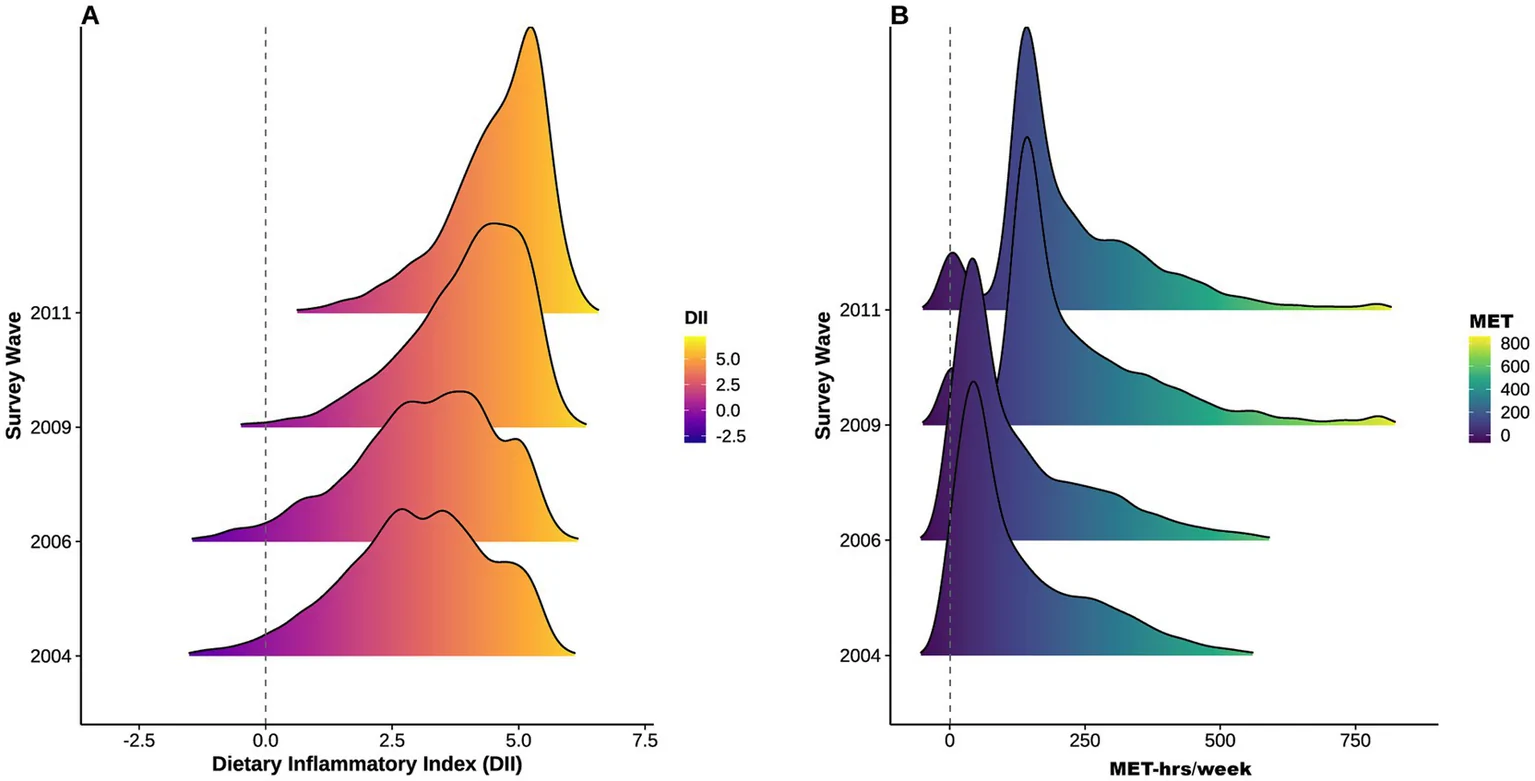
Distributions of DII and physical activity across survey waves from 2004 to 2011. **(A)** Temporal changes in the kernel density distribution of the dietary inflammatory index (DII) across four survey waves from 2004 to 2011. **(B)** Temporal changes in the kernel density distribution of physical activity over the same period. Higher DII scores indicate greater pro-inflammatory dietary potential. Physical activity was expressed as metabolic equivalent hours per week. DII, dietary inflammatory index; MET, metabolic equivalent.

### Identification and characteristics of joint DII–PA trajectory patterns

Joint trajectory models of DII and PA were constructed using four repeated measurements from 2004 to 2011. After comparing model fit indices for two to six trajectory groups, the three-group model was selected as the primary model because it achieved a balanced performance across AIC, BIC, and entropy, with a minimum average posterior probability of 0.750 and a minimum group proportion of 13.6% (Supplementary Figure S2; Supplementary Table S7). The three joint trajectory groups were labeled as lower DII + moderately active (*n* = 559, 14.1%), moderate DII + highly active (*n* = 541, 13.6%), and higher DII + inactive (*n* = 2,870, 72.3%). DII and PA remained clearly stratified across groups during follow-up (Figures 3A,B). The PCA biplot showed that the three groups formed relatively distinguishable clusters in the multidimensional exposure space defined by repeated DII and PA measurements (Figure 3C).

**Figure 3:**
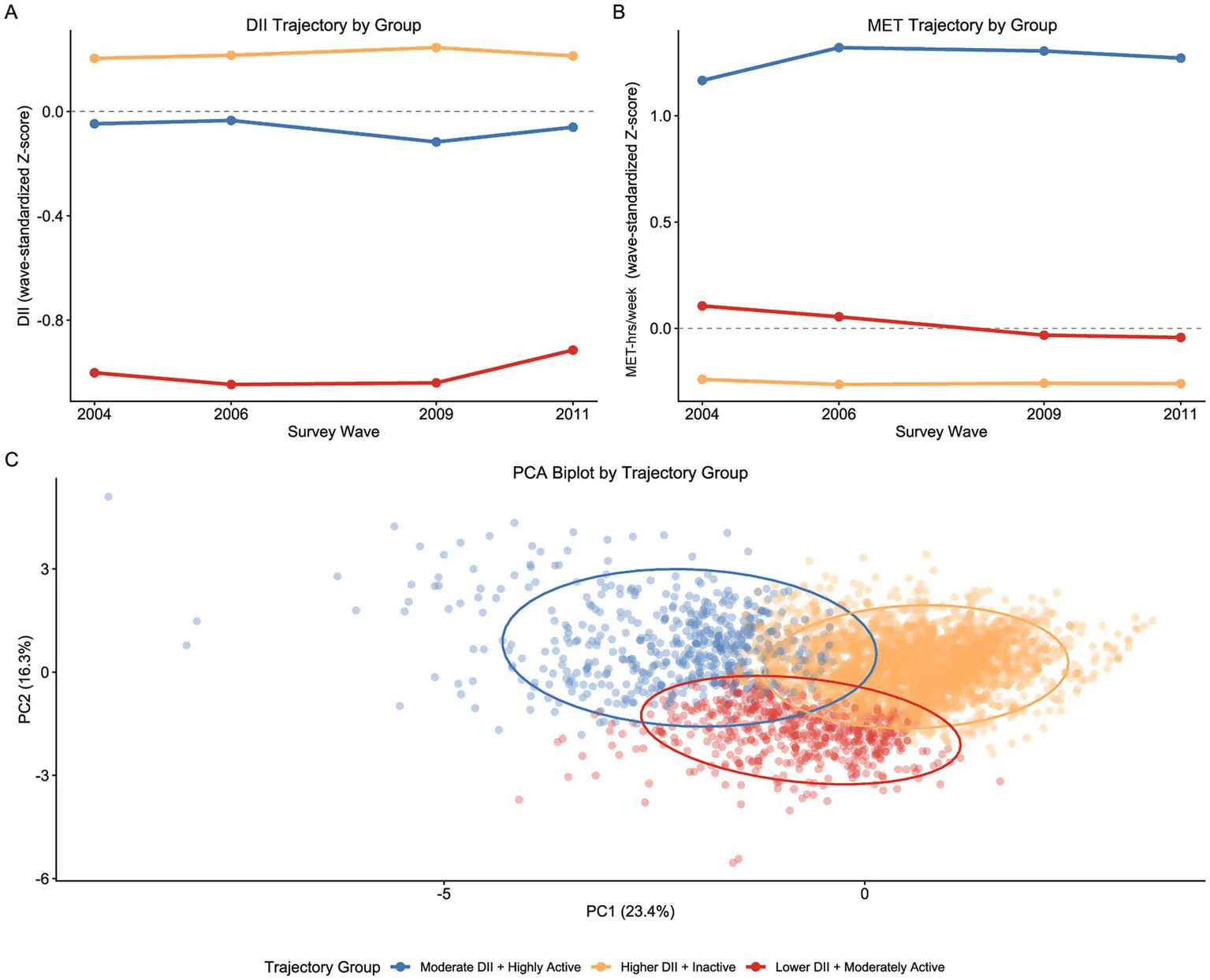
Joint trajectories of DII and physical activity and PCA-based distribution of trajectory groups. **(A)** Longitudinal trajectories of DII across trajectory groups. **(B)** Longitudinal trajectories of physical activity across trajectory groups. Both DII and physical activity were expressed as wave-specific standardized Z scores, and the dashed line at Z = 0 represents the sample mean for each survey wave. A Z score of 0 represents the wave-specific mean; positive and negative values indicate levels above and below the mean, respectively, and the absolute value indicates the distance from the mean in standard deviation units. Higher DII Z scores indicate relatively greater dietary inflammatory potential, whereas higher PA Z scores indicate relatively greater physical activity. **(C)** PCA biplot showing the distribution of trajectory groups in the reduced-dimensional exposure space. Each point represents an individual participant, colors indicate trajectory groups identified by group-based trajectory modeling, and ellipses represent the 95% confidence regions for each trajectory group in the PCA space. DII, dietary inflammatory index; GBTM, group-based trajectory modeling; PCA, principal component analysis; MET, metabolic equivalent.

Baseline characteristics differed across the three trajectory groups (Supplementary Table S8). The lower DII + moderately active group had the lowest DII score (1.6 ± 1.4), a moderate PA level (150.8 ± 115.4 MET-h/week), and higher intakes of total energy, protein, dietary fiber, vitamin C, vitamin E, selenium, and zinc. The moderate DII + highly active group was the youngest (40.3 ± 9.4 years), had the highest proportion of rural residents (76.2%), and showed the highest PA level (297.4 ± 203.3 MET-h/week), with a DII score around the overall mean (2.9 ± 1.4) and intermediate nutritional status. The higher DII + inactive group was the oldest (50.4 ± 14.8 years), had the highest DII score (3.3 ± 1.3), the lowest PA level (103.0 ± 100.2 MET-h/week), the lowest educational attainment, and the lowest intake of multiple nutrients and the highest blood pressure. The prevalence of one baseline CMM component disease was 7.7, 4.1, and 11.8% in the lower DII + moderately active, moderate DII + highly active, and higher DII + inactive groups, respectively (*p* < 0.001). Overall, the three groups represented distinct long-term combinations of dietary inflammatory potential and physical activity.

### Joint trajectory groups and risk of incident CMM

Kaplan–Meier curves showed significant differences in CMM-free survival probability across the three DII–PA joint trajectory groups (log-rank *p* < 0.0001) (Figure 4). The higher DII + inactive group showed the steepest decline in CMM-free survival, whereas the moderate DII + highly active group maintained a relatively higher CMM-free survival probability.

**Figure 4:**
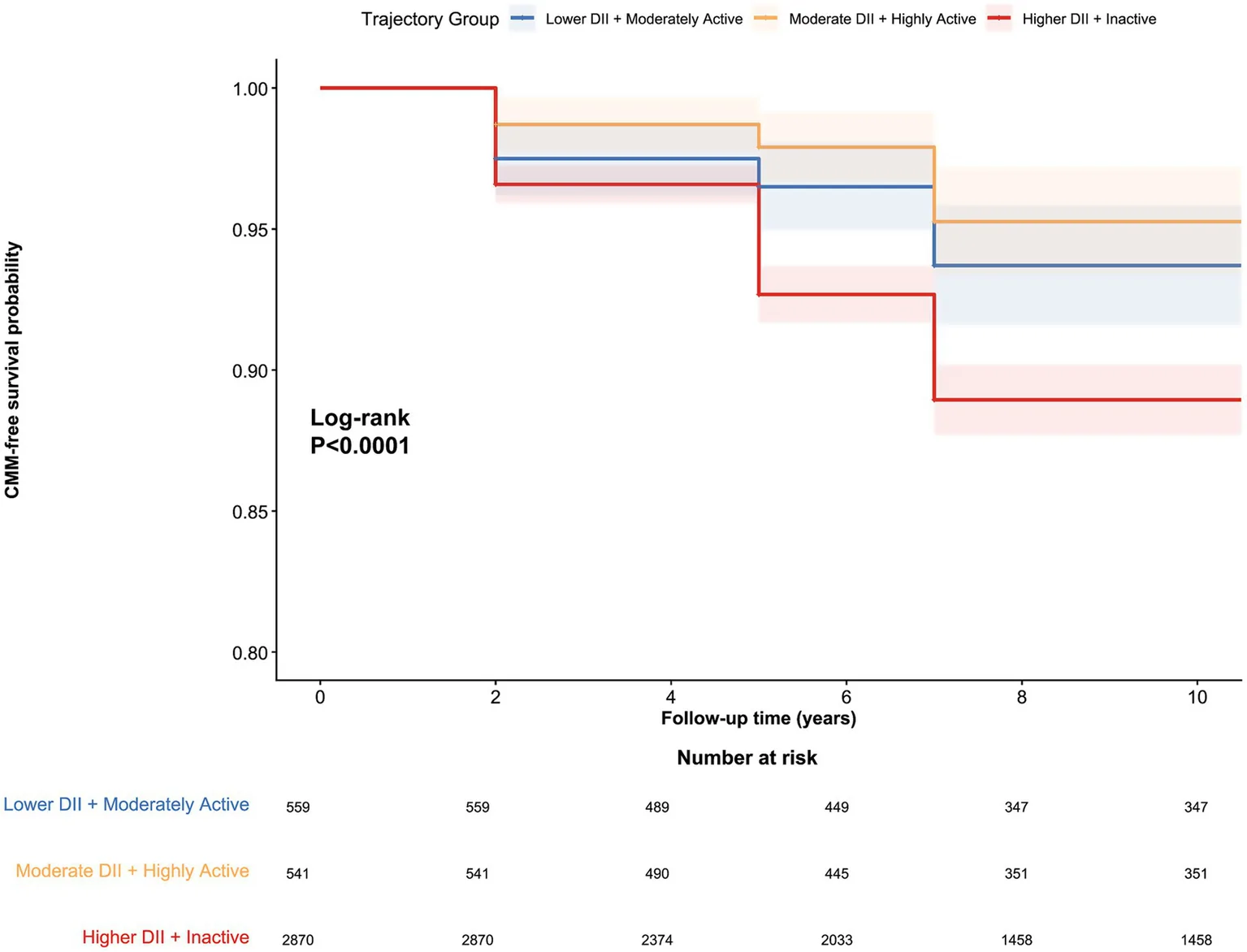
Kaplan–Meier curves for CMM-free survival probability according to joint trajectory group. Survival analyses were stratified by joint trajectory group. Blue represents the lower DII + moderately active group, orange represents the moderate DII + highly active group, and red represents the higher DII + inactive group. Shaded areas indicate 95% confidence intervals. The risk table below the plot shows the number of participants remaining at risk at each time point. Differences in survival curves between groups were assessed using the log-rank test. CMM, cardiometabolic multimorbidity; DII, dietary inflammatory index.

In Cox regression analyses using the lower DII + moderately active group as the reference, the higher DII + inactive group was associated with a significantly increased risk of incident CMM (Table 2). In the unadjusted model, the HR was 1.91 (95% CI: 1.36–2.69). After adjustment for age, residence area, educational level, marital status, BMI, SBP, DBP, baseline CMM component disease status, smoking status, and drinking status, the association remained significant (Model 3: HR = 1.62, 95% CI: 1.14–2.31). In contrast, the moderate DII + highly active group was not associated with a significantly increased risk in any model (all *p* > 0.05). Schoenfeld residual tests showed no evidence of violation of the proportional hazards assumption (global *p* = 0.134) (Supplementary Figure S3).

**Table 2:** Cox regression analysis of joint DII–physical activity trajectory groups and incident CMM risk.

Model	Trajectory group	HR (95% CI)	*p* value
Model 1 (Unadjusted)	Lower DII + Moderately Active (Ref.)	1.00 (Ref.)	Ref.
	Moderate DII + Highly Active	0.87 (0.54–1.4)	0.569
	Higher DII + Inactive	1.91 (1.36–2.69)	<0.001
Model 2 (Demographics)	Lower DII + Moderately Active (Ref.)	1.00 (Ref.)	Ref.
	Moderate DII + Highly Active	1.09 (0.68–1.76)	0.719
	Higher DII + Inactive	1.66 (1.18–2.34)	0.004
Model 3 (Fully adjusted)	Lower DII + Moderately Active (Ref.)	1.00 (Ref.)	Ref.
	Moderate DII + Highly Active	1.13 (0.70–1.84)	0.614
	Higher DII + Inactive	1.62 (1.14–2.31)	0.007

The lower DII + moderately active group was used as the reference. Model 1 was unadjusted. Model 2 was adjusted for age, residence area, educational level, and marital status. Model 3 was further adjusted for body mass index, systolic blood pressure, diastolic blood pressure, baseline CMM component disease status, smoking status and drinking status. Results are presented as HRs with 95% CIs. DII, dietary inflammatory index; CMM, cardiometabolic multimorbidity; HR, hazard ratio; CI, confidence interval.

### Subgroup analyses

Subgroup analyses showed that the increased risk associated with the higher DII + inactive trajectory was statistically significant among participants aged ≥50 years (HR = 1.86, 95% CI: 1.13–3.07), those with primary school education or below (HR = 2.03, 95% CI: 1.23–3.34), those who were married or cohabiting (HR = 1.69, 95% CI: 1.16–2.47), and those without a CMM component disease at baseline (HR = 1.78, 95% CI: 1.21–2.61) (Figure 5). The moderate DII + highly active group was not associated with a significantly increased risk in any subgroup (all *p* > 0.05). No significant interactions were observed for age, education, marital status, or baseline CMM component disease (all *p* for interaction > 0.05), indicating broadly consistent associations across subgroups.

**Figure 5:**
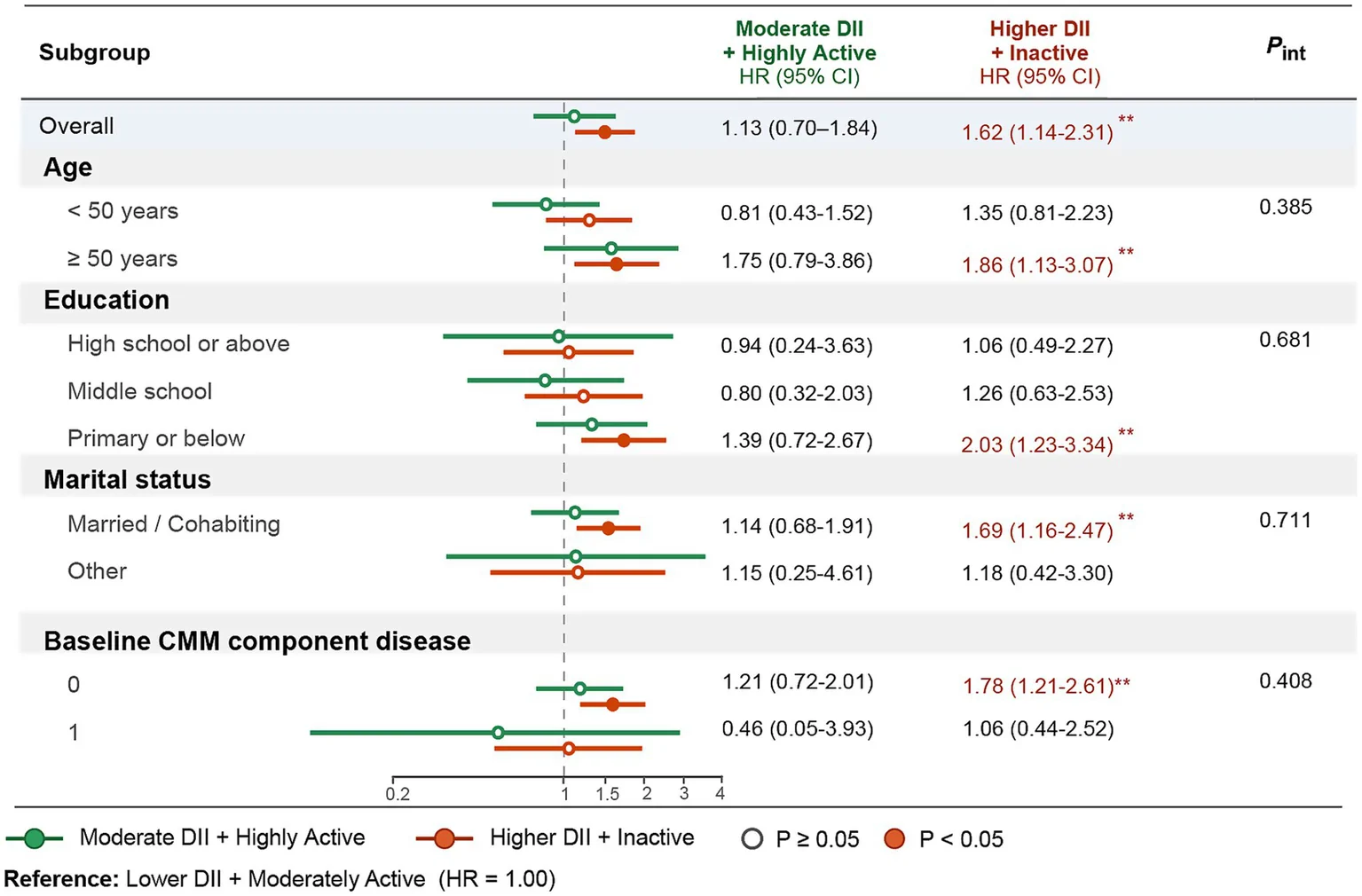
Subgroup analysis of the association between joint trajectory groups and incident CMM. HRs and 95%CIs are presented for the associations of the moderate DII + highly active and higher DII + inactive trajectory groups with incident CMM, using the lower DII + moderately active group as the reference. Subgroups were defined by age (<50 vs. ≥50 years), educational level (high school or above, middle school, and primary school or below), marital status (married/cohabiting vs. other), and baseline CMM component disease status (0 or 1). All models were adjusted for age, residence area, educational level, marital status, body mass index, systolic blood pressure, diastolic blood pressure, baseline CMM component disease status, smoking status, and alcohol consumption status. Effect modification was tested by including interaction terms between trajectory group and subgroup variable in the fully adjusted model. P_int indicates the *p* value for interaction. Solid circles indicate *p* < 0.05, whereas open circles indicate *p* ≥ 0.05. CMM, cardiometabolic multimorbidity; DII, dietary inflammatory index; HR, hazard ratio; CI, confidence interval.

### Sensitivity analyses

The sensitivity analyses yielded results consistent with the primary analysis (Supplementary Table S9). After excluding participants who developed CMM within the first 2 years of follow-up, the higher DII + inactive group remained associated with a higher risk of CMM (HR = 2.02, 95% CI: 1.29–3.17). In the Fine–Gray competing risk model treating death as a competing event, the association remained stable (SHR = 1.62, 95% CI: 1.14–2.29). Similar associations were observed after restricting the analysis to participants with no CMM component disease at baseline (HR = 1.73, 95% CI: 1.19–2.51, *p* = 0.004). The moderate DII + highly active group was not significantly associated with incident CMM in any sensitivity analysis (all *p* > 0.05). These findings support the robustness of the primary results.

## Discussion

Using longitudinal data from the CHNS between 2004 and 2015, this study identified three long-term joint trajectories of DII and PA among adult women. The higher DII + lower PA was significantly associated with an increased risk of incident CMM, whereas the moderate DII + higher PA showed no significant association with CMM risk. These findings suggest that the long-term coexistence of a higher dietary inflammatory burden and insufficient PA may represent an unfavorable lifestyle exposure pattern preceding incident CMM in adult women.

Current cardiometabolic risk screening mainly relies on indicators such as blood pressure, blood glucose, and lipid profiles (22), which often reflect metabolic abnormalities after disease processes have already emerged. Shifting risk assessment upstream to lifestyle factors such as diet and physical activity may help identify long-term behavioral risk patterns before cardiometabolic conditions accumulate into multimorbidity, while also offering greater opportunities for intervention. This approach is consistent with cardiovascular health assessment frameworks (23)and with evidence from prospective studies in China (6, 15). In the present study, women who subsequently developed CMM already had a higher dietary inflammatory burden, lower PA levels, and insufficient intake of several nutrients before disease clustering became apparent. These findings highlight the potential relevance of lifestyle exposures in the early assessment of CMM risk. Consistent with previous studies (24, 25), our baseline analyses also showed that higher DII and lower PA were separately associated with increased CMM risk after multivariable adjustment. No significant additive interaction between DII and PA was observed, while the higher DII + inactive combination remained associated with elevated CMM risk, supporting the joint consideration of both lifestyle exposures and its practical value for risk stratification.

Because both DII and PA change over time, and CMM is a cumulative outcome, a single cross-sectional measurement may not adequately capture long-term exposure patterns. By applying joint trajectory analysis, this study extended baseline exposure assessment to longitudinal exposure patterns and identified three trajectory groups that remained clearly stratified during follow-up. Notably, the higher DII + inactive trajectory was associated with an increased risk of CMM. Women in this trajectory group also tended to be older, had less favorable nutritional profiles, and more frequently had one CMM component disease at baseline. Importantly, the association remained after adjustment for BMI, blood pressure, and baseline CMM component disease status and in the sensitivity analysis restricted to women free of all four component diseases at baseline. These findings reduce the likelihood that the observed association was driven primarily by pre-existing cardiometabolic disease burden at study entry. From a pathophysiological perspective, the component diseases of CMM share interrelated processes, including chronic inflammation, insulin resistance, vascular inflammation, and endothelial dysfunction (26, 27). Higher dietary inflammatory burden and insufficient PA may contribute to chronic inflammatory, metabolic, and vascular dysregulation over time, while an unfavorable nutritional profile may further contribute to cardiometabolic risk (28). In contrast, the long-term pattern of moderate DII combined with higher PA was not associated with a clear increase in risk, which may be partly related to differences in demographic and lifestyle background in this group. Together, these findings indicate that long-term joint DII–PA patterns capture sustained lifestyle exposure profiles associated with CMM risk and extend the information provided by baseline exposure assessment.

Previous evidence suggests that a favorable lifestyle may reduce cardiometabolic disease-related mortality by approximately 63% (29), and women may derive stronger and more durable benefits from low cardiovascular risk profiles (30). Therefore, incorporating DII and PA into long-term cardiometabolic risk monitoring may provide additional information for the early prevention of CMM in women. Notably, the association between the higher DII + inactive trajectory and incident CMM remained evident among women free of all four CMM component diseases at baseline, extending the relevance of this lifestyle pattern to an earlier stage of cardiometabolic disease development. Women with a higher DII + lower PA trajectory may face a higher long-term risk of CMM and warrant closer attention in primary care and community-based follow-up. These implications are particularly relevant in the Chinese context. Chinese women often take primary responsibility for household food purchasing and meal preparation, meaning that their dietary behaviors may affect not only their own health but also the nutritional health of the entire family (31). Meanwhile, physical activity among middle-aged and older women is often embedded in occupational work, household caregiving, and daily routines, and is strongly constrained by time burden and access to community resources (32). Integrated lifestyle interventions may therefore simultaneously promote healthier household dietary patterns and incorporate physical activity into daily contexts such as commuting and housework, thereby improving the feasibility of behavioral change. Overall, the DII–PA joint trajectories identified in this study deepen the understanding of long-term lifestyle patterns associated with CMM and may inform lifestyle-oriented prevention in community and primary care settings.

### Limitations

This study has several limitations. First, as an observational cohort study, residual confounding and reverse causation cannot be fully excluded, although multivariable adjustment and sensitivity analyses excluding early CMM events and restricting analyses to women free of all four CMM components at baseline. Some participants may have had subclinical metabolic abnormalities before baseline or before CMM was formally ascertained during follow-up, which could have influenced subsequent dietary and physical activity patterns. Second, the CHNS did not consistently collect information on menopausal status. Adjustment for age can only partially account for metabolic changes related to the female life course, which represents an unavoidable limitation of research focused on women. Information on medication use, family history, sleep, and psychological factors was also unavailable, limiting the scope of confounding control. Third, PA was assessed using self-reported questionnaires and was therefore subject to recall bias; the questionnaire also could not precisely distinguish differences in the composition of occupational, household, and leisure-time activities. Fourth, CMM components were primarily ascertained from self-reported physician diagnoses, while objective measurements were not consistently available across all component diseases and survey waves, which may have introduced disease misclassification. In addition, the exact onset time of CMM between consecutive survey waves could not be determined, reducing the temporal precision of event ascertainment. Fifth, data availability decreased across follow-up waves, and participants included in the final analysis differed from those excluded in several baseline characteristics, indicating the possibility of selection bias related to incomplete follow-up or exposure data. Finally, the study population was derived from a single cohort, and the external generalizability of the findings requires validation in other Chinese female populations. Future studies should incorporate objective PA measurements, repeated objective cardiometabolic measurements, menopausal status and reproductive history, medication use, and inflammatory biomarkers in multicenter prospective cohorts to better control for key confounders, and assess the reproducibility and external applicability of the observed associations.

## Conclusion

This study showed that, before the development of CMM, adult women already exhibited distinct long-term combinations of dietary inflammatory burden and PA. By constructing long-term joint trajectories of DII and PA using repeated measurements, this study identified an association between longitudinal behavioral exposure patterns and incident CMM. In particular, the persistent coexistence of a more pro-inflammatory diet and lower physical activity was associated with an increased risk of CMM. These findings highlight the relevance of sustained dietary and PA patterns to CMM development even at an early stage without established cardiometabolic disease, and support lifestyle-oriented prevention at an earlier stage among adult women in China.

## Data Availability

The original contributions presented in the study are included in the article/Supplementary material, further inquiries can be directed to the corresponding author.

## Supplementary material

SUPPLEMENTARY FIGURE S1 Restricted cubic spline analyses of baseline DII and physical activity with incident CMM risk. **(A)** Association between baseline DII and incident CMM risk. **(B)** Association between baseline total physical activity and incident CMM risk. Solid lines indicate HRs, shaded areas indicate 95% CIs, and the horizontal dashed line indicates HR = 1. DII, dietary inflammatory index; CMM, cardiometabolic multimorbidity; HR, hazard ratio; CI, confidence interval; MET, metabolic equivalent.

SUPPLEMENTARY FIGURE S2 Model selection criteria for joint DII–physical activity trajectory models. Model fit and classification quality were compared across joint trajectory models with two to six groups, with the final three-group model highlighted by a red circle. **(A)** Changes in BIC across models with different numbers of trajectory groups. **(B)** Minimum average posterior probability across models; the dashed line indicates the prespecified threshold of 0.70. **(C)** Minimum group proportion across models; the dashed line indicates the prespecified threshold of 5%. **(D)** Entropy across models. DII, dietary inflammatory index; BIC, Bayesian information criterion.

SUPPLEMENTARY FIGURE S3 Schoenfeld residual tests for the proportional hazards assumption in the Cox model. Scaled Schoenfeld residuals over time are shown for joint trajectory group and each covariate in the fully adjusted Cox model. Circles indicate scaled Schoenfeld residuals, solid lines indicate smoothed residual trends, and dashed lines indicate 95% confidence intervals. A nonsignificant time trend indicates no clear evidence of violation of the proportional hazards assumption. The Cox model included joint trajectory group, age, educational level, marital status, body mass index, systolic blood pressure, diastolic blood pressure, baseline CMM component disease status, smoking status, and drinking status. DII, dietary inflammatory index; CMM, cardiometabolic multimorbidity.

SUPPLEMENTARY Table S1 Dietary parameters, global reference values, and inflammatory effect scores used for DII calculation. This table lists the 24 dietary parameters used to calculate the DII, including 18 nutrient-level parameters and six food-level parameters. Global_Mean and Global_SD indicate the mean and standard deviation of each parameter in the DII global reference database, respectively. Effect_Score indicates the literature-derived inflammatory effect score for each dietary parameter. Direction indicates the direction of contribution to the DII score, with “+” indicating a pro-inflammatory contribution and “−” indicating an anti-inflammatory contribution. Available_Waves indicates the survey years in which the parameter was available in the CHNS. DII, dietary inflammatory index; CHNS, China Health and Nutrition Surve.

SUPPLEMENTARY Table S2 Variable Definitions and Disease Ascertainment Criteria Used in the CHNS. Definitions and source variables are provided for the four CMM components and other study variables. CMM was defined as the coexistence of at least two of hypertension, diabetes, stroke, and heart disease. Heart disease was ascertained using available CHNS heart disease and myocardial infarction items. Chronic diseases (hypertension, diabetes, heart disease, stroke) are treated as irreversible conditions. Once reported at any survey wave, all subsequent waves are coded as positive regardless of reporting status. Dietary data used for DII calculation were obtained from the 2004, 2006, 2009, and 2011 survey waves. CMM, cardiometabolic multimorbidity; MI, myocardial infarction; SBP, systolic blood pressure; DBP, diastolic blood pressure; BMI, body mass index; DII, dietary inflammatory index; MET, metabolic equivalent; NA, not applicable.

SUPPLEMENTARY Table S3 Missingness of key variables by survey wave among the included analytic sample (N=3970). Available and missing observations are presented as numbers and percentages using the analytic sample (N = 3,970) as the denominator. CMM outcome data were considered available when sufficient disease information was present to determine CMM status at the corresponding survey wave. Heart disease was ascertained using the available CHNS heart disease and myocardial infarction items. CMM, cardiometabolic multimorbidity; DII, dietary inflammatory index; PA, physical activity; BMI, body mass index; SBP, systolic blood pressure; DBP, diastolic blood pressure; MET, metabolic equivalent.

SUPPLEMENTARY Table S4 Baseline characteristics of included and excluded participants. Baseline characteristics were compared between participants included in the analytic cohort (n = 3,970) and those excluded because of incomplete follow-up or insufficient longitudinal exposure data (n = 599). Continuous variables are presented as means ± standard deviations and categorical variables as numbers (percentages). BMI, body mass index; WHtR, waist-to-height ratio; SBP, systolic blood pressure; DBP, diastolic blood pressure; DII, dietary inflammatory index; PA, physical activity; MET, metabolic equivalent; CMM, cardiometabolic multimorbidity.

SUPPLEMENTARY Table S5 Associations of baseline DII and physical activity tertiles with incident CMM. Baseline DII and total physical activity were separately categorized into tertiles, and Cox proportional hazards models were fitted for each exposure. The lowest DII tertile was used as the reference in the DII analysis, and the lowest physical activity tertile was used as the reference in the physical activity analysis. Results are presented as hazard ratios (HRs) with 95% confidence intervals (CIs). Unadjusted analyses included 3,970 participants with 400 events; fully adjusted analyses included 3,895 participants with 392 events because complete baseline blood pressure data were required. Fully adjusted models included age, residence area, educational level, marital status, body mass index, systolic blood pressure, diastolic blood pressure, baseline CMM component disease status, smoking status, and drinking status. DII, dietary inflammatory index; CMM, cardiometabolic multimorbidity; HR, hazard ratio; CI, confidence interval; MET, metabolic equivalent.

SUPPLEMENTARY Table S6 Additive interaction between baseline DII and physical activity on incident CMM risk. Baseline DII and total physical activity were dichotomized at their respective medians and cross-classified into four joint exposure groups. DII was dichotomized at the median of 3.07, and total physical activity was dichotomized at the median of 82.8 MET-h/week. The lower DII + active group was used as the reference. Additive interaction was evaluated using the relative excess risk due to interaction (RERI), AP, and SI, with 95% CIs estimated using 500 bootstrap resamples. Models were adjusted for age, residence, education, marital status, body mass index, systolic blood pressure, diastolic blood pressure, baseline CMM component disease status, smoking, and alcohol consumption. RERI > 0 indicates positive additive interaction. DII, dietary inflammatory index; CMM, cardiometabolic multimorbidity; HR, hazard ratio; CI, confidence interval; RERI, relative excess risk due to interaction; AP, attributable proportion due to interaction; SI, synergy index.

SUPPLEMENTARY Table S7 Model selection criteria for joint trajectory models. Model fit and classification quality were compared across joint trajectory models with two to six groups. AIC indicates Akaike information criterion; BIC, Bayesian information criterion; Log-Likelihood, model log-likelihood; Min AvePP, the lowest average posterior probability across trajectory groups; Min Group %, the proportion of the smallest trajectory group in the overall sample; and Entropy, classification clarity. The final model was selected by jointly considering model fit, average posterior probability, minimum group proportion, and interpretability of the trajectory patterns. DII, dietary inflammatory index; PA, physical activity; AIC, Akaike information criterion; BIC, Bayesian information criterion; AvePP, average posterior probability.

SUPPLEMENTARY Table S8 Baseline characteristics of study participants by joint trajectory group. Continuous variables are presented as means ± standard deviations, and categorical variables are presented as numbers with percentages. The DII–PA joint trajectory groups included lower DII + moderately active, moderate DII + highly active, and higher DII + inactive. Higher DII scores indicate greater pro-inflammatory dietary potential. Physical activity was expressed as metabolic equivalent hours per week. Between-group comparisons were performed using analysis of variance or the rank-sum test for continuous variables and the χ² test for categorical variables. PA, physical activity; DII, dietary inflammatory index; BMI, body mass index; WHtR, waist-to-height ratio; SBP, systolic blood pressure; DBP, diastolic blood pressure; MET, metabolic equivalent.

SUPPLEMENTARY Table S9 Sensitivity analyses of the association between joint trajectories and incident CMM. Three sensitivity analyses were conducted: (1) Cox proportional hazards regression after excluding participants who developed CMM within the first two years of follow-up; (2) Fine–Gray competing risk models treating death as a competing event; and (3) Cox regression restricted to participants free of all four CMM component diseases at baseline. Cox regression results are presented as hazard ratios (HRs) with 95% confidence intervals (CIs), and Fine–Gray model results are presented as subdistribution hazard ratios (SHRs) with 95% CIs. All analyses used the Model 3 covariate set; baseline CMM component disease status was omitted from the third analysis because it was invariant in the restricted sample. Model 3 were adjusted for age, residence area, educational level, marital status, body mass index, systolic blood pressure, diastolic blood pressure, baseline CMM component disease status, smoking status, and drinking status. DII, dietary inflammatory index; CMM, cardiometabolic multimorbidity; HR, hazard ratio; SHR, subdistribution hazard ratio; CI, confidence interval.
